# A novel case of cutaneous plasmacytosis in a patient of Native American ancestry

**DOI:** 10.1016/j.jdcr.2025.11.032

**Published:** 2025-11-29

**Authors:** Giovanna A. Miller, Tyler Enos, Scott D. Miller

**Affiliations:** a Amarillo College, Amarillo, Texas; b High Plains Dermatology Center, Amarillo, Texas; c Clinical Assistant Professor Texas Tech Health Sciences Center, Amarillo, Texas

**Keywords:** cutaneous plasmacytosis, general dermatology, hematology, medical dermatology

## Introduction

Cutaneous plasmacytosis is a rare condition of unknown etiology that occurs predominantly in patients of Southeast Asian descent and is characterized by the benign proliferation of polyclonal plasma cells. It typically presents as reddish-brown papules and plaques, most commonly affecting the trunk and face.^1^ In the literature, we found only 11 reported cases in individuals of non-Southeast Asian descent. Here, we report another rare exception: a 59-year-old male with Native American ancestry.

## Case report

**Fig 1:**
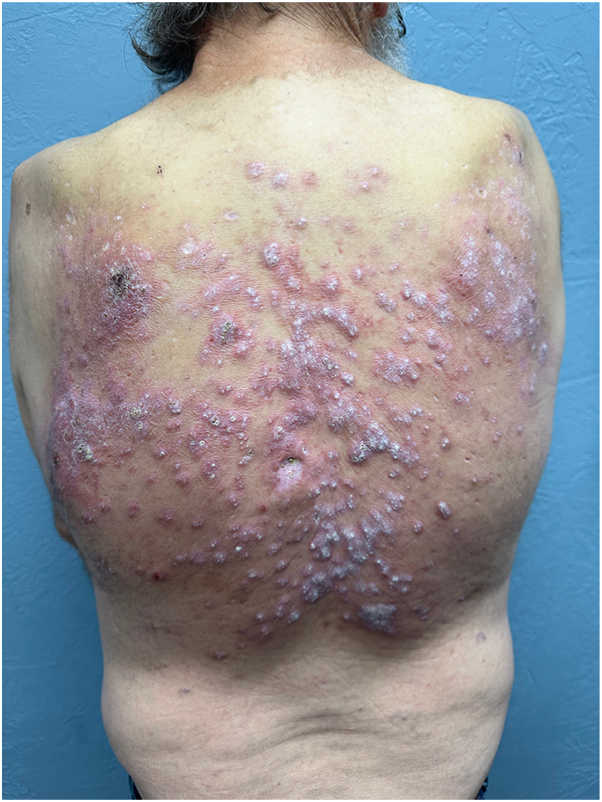
Cutaneous plasmacytosis presenting as reddish papules and plaques on the patient’s back.

**Fig 2:**
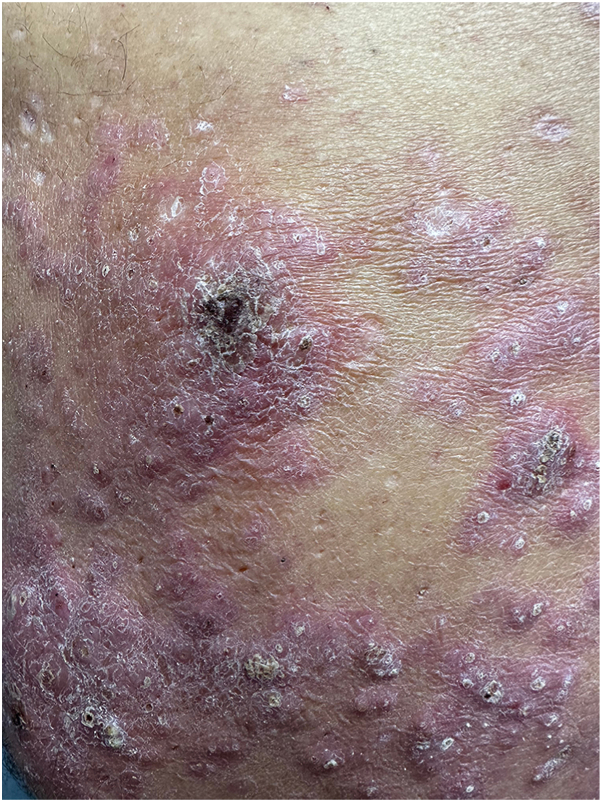
Close-up view of cutaneous plasmacytosis.

**Fig 3:**
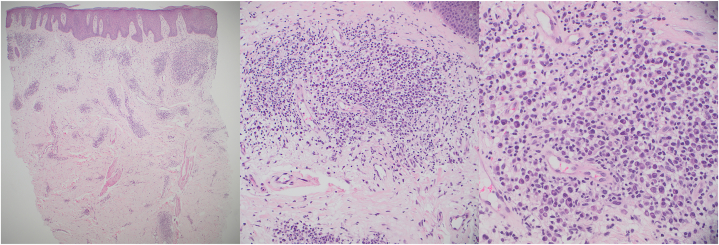
Hematoxylin and eosin–stained section showing a superficial and deep perivascular infiltrate composed of lymphocytes, histiocytes, and numerous mature plasma cells. The left portion of the image is shown at 40× magnification, with 200× in the center and 400× on the right.

**Fig 4:**
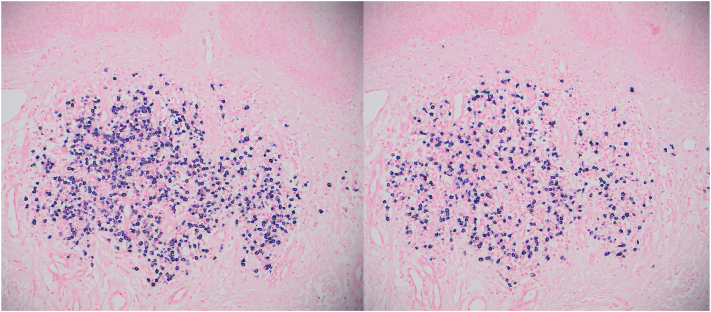
Kappa (shown on the left at 100× magnification) and lambda (shown on the right) in situ hybridization studies reveal a ratio of approximately 2:1.

A 59-year-old male of mixed Occidental and Native American descent presented with a 20-year history of generally asymptomatic, reddish to violaceous hypertrophic papules and plaques on his back (Figs 1 and 2). He was otherwise in good health, without fever, chills, malaise, or weight loss. No evidence of polyneuropathy or organomegaly was found, and he had no history of travel outside the continental United States. Over the years, numerous providers had treated his rash with various therapies, including topical glucocorticosteroids of different strengths, oral antibiotics such as doxycycline, antimalarial drugs such as hydroxychloroquine, dupilumab, and oral isotretinoin. According to the patient, none of these treatments provided any benefit. Skin examination revealed nontender, reddish to violaceous hypertrophic papules and plaques covering most of the back, without associated palpable lymphadenopathy. A punch biopsy was performed to further characterize the eruption, showing a superficial and deep perivascular infiltrate composed of lymphocytes and numerous mature plasma cells without atypia (Fig 3). These cells were positive for cluster of differentiation 138. Kappa and lambda in situ hybridization revealed a ratio of approximately 2:1 (Fig 4). The immunoglobulin G4 to immunoglobulin G ratio was less than 20%. A spirochete immunohistochemical stain for organisms was negative.

Laboratory workup included a complete blood count, protein electrophoresis, urinalysis, rapid plasma reagin, and HIV antibody testing, all of which were largely unremarkable, except for a mild elevation of gamma globulin at 2.1 (reference range: 0.4-1.8).

## Discussion

We describe a patient with Native American ancestry who presented with a rare condition, cutaneous plasmacytosis, typically reported almost exclusively in individuals of Southeast Asian descent. The condition was first described in 1976 by Yashiro^1^ and later further characterized by Kitamura et al^2^ in 1980 as reddish-brown papules and plaques, usually favoring the trunk and face, composed of polyclonal plasma cells, and generally following a benign, chronic course. We note that our patient’s lesions were noticeably less brown and more hypertrophic than is typically described in the literature, which may in part be explained by his phenotype being atypical for this disease. Cutaneous plasmacytosis has been reported with a male-to-female ratio of 1:0.6, at a mean and median age of 37 years (range: 20-62 years).^3^

Plasmacytosis should be differentiated from a multitude of other plasma cell disorders. Syphilis can be excluded by negative treponemal immunohistochemistry and rapid plasma reagin tests. A plasma cell variant of multicentric Castleman’s disease is characterized by sheets of plasma cells within lymph nodes, often associated with systemic symptoms like fever and organ dysfunction secondary to hypercytokinemia. Multicentric Castleman’s disease’s constellation of findings were absent in our patient’s presentation. POEMS (polyneuropathy, organomegaly, endocrinopathy, monoclonal protein, and skin changes) and AESOP (adenopathy and extensive skin patch overlying a plasmacytoma) syndromes can be ruled out by the absence of their characteristic findings, including polyneuropathy, organomegaly, endocrinopathy, bone involvement, and life-threatening progression. Similarly, the distribution pattern excludes other benign mucocutaneous plasma cell disorders, such as plasma cell gingivitis, plasma cell cheilitis, Zoon’s balanitis, and Zoon’s vulvitis. The etiology of cutaneous plasmacytosis remains undefined. Some speculate that upstream signaling molecules may influence interleukin-6 activity, which drives B-cell differentiation into plasma cells, in a manner similar to cytokine effects observed in Castleman disease.^4^

Treatment outcomes tend to be disappointing, as observed in our patient. Reported therapies are extensive and include systemic, intralesional, and topical corticosteroids; topical tacrolimus; antibiotics; systemic chemotherapies such as melphalan; anti-CD20 antibody therapy; prednisone–cyclophosphamide combination therapy; radiotherapy; psoralen and UV-A light; and thalidomide.^5^, ^6^, ^7^, ^8^, ^9^

Plasmacytosis is an extremely rare disease with little known about its causes or effective treatments. Identification of additional cases may help further characterize this unusual process. We present this unique occurrence in the hope that its contribution to the epidemiological literature may aid in the future delineation and definition of this elusive disease.

## Conflicts of interest

None disclosed.
